# Tetra­kis(1-allyl-1*H*-imidazole-κ*N* ^3^)bis­(thio­cyanato-κ*N*)manganese(II)

**DOI:** 10.1107/S1600536811051282

**Published:** 2011-12-03

**Authors:** Juan Zhao, Yan-Ling Jin

**Affiliations:** a College of Mechanical Engineering, Qingdao Technological University, Qingdao 266033, People’s Republic of China; b Key Laboratory of Advanced Materials, Qingdao University of Science and Technology, Qingdao 266042, People’s Republic of China

## Abstract

The structure of the title compound, [Mn(NCS)_2_(C_6_H_8_N_2_)_4_], consists of isolated mol­ecules of [Mn(NCS)_2_(Aim)_4_] (Aim = 1-allyl­imidazole), which contain a compressed octa­hedral MnN_6_ chromophore (site symmetry 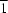). The NCS^−^ anions are *trans* and four N atoms from the Aim ligands define the equatorial plane. The mean Mn—N(Aim) and Mn—N(NCS) distances are 2.270 and 2.229 Å, respectively. Weak C—H⋯N inter­actions contribute to the crystal packing stability.

## Related literature

In the corresponding manganese compound [Mn(NCS)_2_(1-ethyl­imidazole)_4_] (Liu, *et al.*, 2008 ▶), the Mn^II^ ions have a distorted octa­hedral environment.
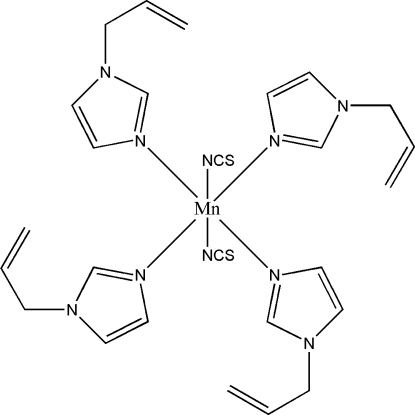


## Experimental

### 

#### Crystal data

[Mn(NCS)_2_(C_6_H_8_N_2_)_4_]
*M* *_r_* = 603.70
Monoclinic, 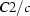

*a* = 24.564 (5) Å
*b* = 7.2200 (14) Å
*c* = 21.287 (4) Å
β = 125.04 (3)°
*V* = 3091.0 (15) Å^3^
*Z* = 4
Mo *K*α radiation
μ = 0.60 mm^−1^
*T* = 293 K
0.20 × 0.10 × 0.10 mm

#### Data collection

Bruker SMART 1K CCD area-detector diffractometer
Absorption correction: multi-scan (*SADABS*; Sheldrick, 2004 ▶) *T*_min_ = 0.890, *T*_max_ = 0.943
2885 measured reflections
2814 independent reflections
1750 reflections with *I* > 2σ(*I*)
*R*_int_ = 0.033

#### Refinement

*R*[*F* ^2^ > 2σ(*F* ^2^)] = 0.059
*wR*(*F* ^2^) = 0.162
*S* = 1.01
2814 reflections
178 parameters
H-atom parameters constrained
Δρ_max_ = 0.31 e Å^−3^
Δρ_min_ = −0.34 e Å^−3^

### 

Data collection: *SMART* (Bruker, 2001 ▶); cell refinement: *SAINT* (Bruker, 2001 ▶); data reduction: *SAINT*; program(s) used to solve structure: *SHELXTL* (Sheldrick, 2008 ▶); program(s) used to refine structure: *SHELXTL*; molecular graphics: *SHELXTL*; software used to prepare material for publication: *SHELXTL* and local programs.

## Supplementary Material

Crystal structure: contains datablock(s) global, I. DOI: 10.1107/S1600536811051282/hg5141sup1.cif

Structure factors: contains datablock(s) I. DOI: 10.1107/S1600536811051282/hg5141Isup2.hkl

Supplementary material file. DOI: 10.1107/S1600536811051282/hg5141Isup3.cdx

Additional supplementary materials:  crystallographic information; 3D view; checkCIF report

## Figures and Tables

**Table 1:** Hydrogen-bond geometry (Å, °)

*D*—H⋯*A*	*D*—H	H⋯*A*	*D*⋯*A*	*D*—H⋯*A*
C7—H7*A*⋯N3	0.93	2.54	2.857 (9)	101
C6—H6*A*⋯N4	0.93	2.88	3.355 (8)	113
C5—H5*B*⋯N4^i^	0.93	2.82	3.298 (7)	113
C12—H12*A*⋯N5^i^	0.93	2.72	3.224 (6)	115

Symmetry code: (i) 
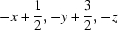
.

**Fig. 1.**
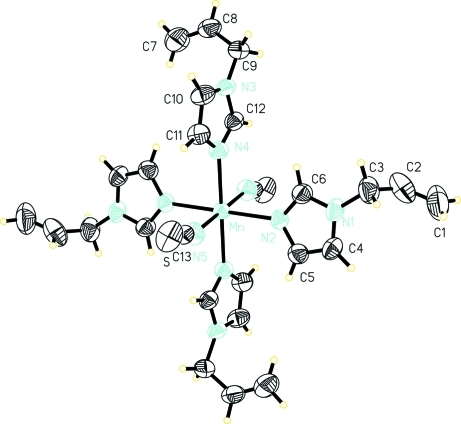
The structure of the title compound, showing 50% probability displacement ellipsoids and the atom-numbering scheme.

**Fig. 2.**
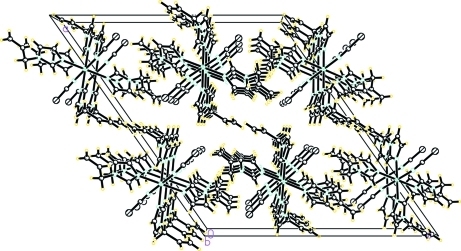
The packing of (I), viewed down the b axis.

## supplementary crystallographic information

### Comment

The molecular structure of (I) is shown in Fig. 1. The Mn atom displays an octahedral coordination geometry, with six N atoms from two thiocyanate anions and four 1-allylimidazole ligands. The equatorial plane of the complex is formed by four Mn—*N*(1-allylimadazole) bonds with lengths of 2.269 (3) and 2.271 (3) Å, and the axial positions are occupied by two N-bonded NCS groups [Mn—N(NCS) = 2.229 (4) Å]. These values agree well with those observed in [Mn(NCS)_2_(1-ethylimidazole)_4_] (Liu *et al.*, 2008). The values of the bond angles around manganese are close to those expected for a regular octahedral geometry, the N—Mn—N angles range from 88.32 (13) to 91.68 (13) °, and the thiocyanate ligands are almost linear. Weak C—H···N interactions contribute to the crystal packing stability.

In the corresponding manganese compound [Mn(NCS)_2_(1-ethylimidazole)_4_] (Liu, *et al.*, 2008), the Mn^II^ ions have a distorted octahedral environment.

### Experimental

The title compound was prepared by the reaction of 1-allylimidazole (1.21 g, 20 mmol) with MnCl_2_.4H_2_O(0.99 g, 5 mmol) and potassium thiocyanate (0.98 g, 10 mmol) by means of hydrothermal synthesis in stainless-steel reactor with Teflon liner at 383 K for 24 h. Analysis, calculated for C_26_H_32_MnN_10_S_2_: C 51.73, H 5.34, N 23.20%; found: C 51.97, H 5.29, N 23.01%. Single crystals suitable for X-ray measurements were obtained by recrystallization from methanol at room temperature.

### Refinement

H atoms were positioned geometrically(C—H = 0.93–0.97 Å) and allowed to ride on their parent atoms with *U*_iso_(H) = 1.2 times *U*_eq_(C).

### Crystal data

**Table:**

[Mn(NCS)_2_(C_6_H_8_N_2_)_4_]	*F*(000) = 1260
*M**_r_* = 603.70	*D*_x_ = 1.297 Mg m^−^^3^
Monoclinic, *C*2/*c*	Mo *K*α radiation, λ = 0.71073 Å
Hall symbol: -C 2yc	Cell parameters from 25 reflections
*a* = 24.564 (5) Å	θ = 9–12°
*b* = 7.2200 (14) Å	µ = 0.60 mm^−^^1^
*c* = 21.287 (4) Å	*T* = 293 K
β = 125.04 (3)°	Block, colorless
*V* = 3091.0 (15) Å^3^	0.20 × 0.10 × 0.10 mm
*Z* = 4	

### Data collection

**Table:**

Bruker SMART 1K CCD area-detector diffractometer	2814 independent reflections
Radiation source: fine-focus sealed tube	1750 reflections with *I* > 2σ(*I*)
graphite	*R*_int_ = 0.033
thin–slice ω scans	θ_max_ = 25.3°, θ_min_ = 2.0°
Absorption correction: multi-scan (*SADABS*; Sheldrick, 2004)	*h* = 0→29
*T*_min_ = 0.890, *T*_max_ = 0.943	*k* = 0→8
2885 measured reflections	*l* = −25→20

### Refinement

**Table:**

Refinement on *F*^2^	Primary atom site location: structure-invariant direct methods
Least-squares matrix: full	Secondary atom site location: difference Fourier map
*R*[*F*^2^ > 2σ(*F*^2^)] = 0.059	Hydrogen site location: inferred from neighbouring sites
*wR*(*F*^2^) = 0.162	H-atom parameters constrained
*S* = 1.01	*w* = 1/[σ^2^(*F*_o_^2^) + (0.075*P*)^2^] where *P* = (*F*_o_^2^ + 2*F*_c_^2^)/3
2814 reflections	(Δ/σ)_max_ < 0.001
178 parameters	Δρ_max_ = 0.31 e Å^−^^3^
0 restraints	Δρ_min_ = −0.34 e Å^−^^3^

### Special details

**Table:**

Geometry. All e.s.d.'s (except the e.s.d. in the dihedral angle between two l.s. planes) are estimated using the full covariance matrix. The cell e.s.d.'s are taken into account individually in the estimation of e.s.d.'s in distances, angles and torsion angles; correlations between e.s.d.'s in cell parameters are only used when they are defined by crystal symmetry. An approximate (isotropic) treatment of cell e.s.d.'s is used for estimating e.s.d.'s involving l.s. planes.
Refinement. Refinement of *F*^2^ against ALL reflections. The weighted *R*-factor *wR* and goodness of fit *S* are based on *F*^2^, conventional *R*-factors *R* are based on *F*, with *F* set to zero for negative *F*^2^. The threshold expression of *F*^2^ > σ(*F*^2^) is used only for calculating *R*-factors(gt) *etc*. and is not relevant to the choice of reflections for refinement. *R*-factors based on *F*^2^ are statistically about twice as large as those based on *F*, and *R*- factors based on ALL data will be even larger.

### Fractional atomic coordinates and isotropic or equivalent isotropic displacement parameters (Å^2^)

**Table:**

	*x*	*y*	*z*	*U*_iso_*/*U*_eq_	
Mn	0.2500	0.7500	0.0000	0.0393 (3)	
S	0.38490 (7)	1.1290 (2)	0.23301 (7)	0.0754 (5)	
N1	0.20466 (18)	0.4508 (6)	0.1457 (2)	0.0542 (10)	
N2	0.21204 (16)	0.6197 (5)	0.06496 (19)	0.0447 (9)	
N3	0.41287 (16)	0.3410 (5)	0.0976 (2)	0.0457 (9)	
N4	0.34393 (16)	0.5759 (5)	0.06687 (19)	0.0448 (9)	
N5	0.29713 (18)	0.9709 (5)	0.0893 (2)	0.0543 (10)	
C1	0.1320 (3)	0.1161 (10)	0.1924 (4)	0.116 (3)	
H1A	0.1358	0.1802	0.2327	0.139*	
H1B	0.1016	0.0196	0.1686	0.139*	
C2	0.1692 (3)	0.1613 (9)	0.1695 (3)	0.0911 (19)	
H2A	0.1640	0.0938	0.1291	0.109*	
C3	0.2181 (3)	0.3078 (8)	0.2018 (3)	0.0725 (16)	
H3A	0.2616	0.2548	0.2227	0.087*	
H3B	0.2191	0.3649	0.2437	0.087*	
C4	0.1559 (2)	0.5784 (7)	0.1165 (3)	0.0570 (13)	
H4A	0.1250	0.5921	0.1281	0.068*	
C5	0.1607 (2)	0.6819 (7)	0.0675 (3)	0.0540 (12)	
H5B	0.1332	0.7812	0.0394	0.065*	
C6	0.2375 (2)	0.4801 (7)	0.1133 (3)	0.0533 (12)	
H6A	0.2736	0.4103	0.1238	0.064*	
C7	0.4849 (3)	0.2836 (9)	0.0313 (3)	0.0841 (18)	
H7A	0.4652	0.3981	0.0257	0.101*	
H7B	0.5106	0.2656	0.0126	0.101*	
C8	0.4765 (2)	0.1509 (8)	0.0647 (3)	0.0626 (14)	
H8A	0.4972	0.0392	0.0689	0.075*	
C9	0.4373 (2)	0.1569 (7)	0.0974 (3)	0.0577 (13)	
H9A	0.3996	0.0740	0.0683	0.069*	
H9B	0.4647	0.1113	0.1497	0.069*	
C10	0.4487 (2)	0.4823 (7)	0.1465 (3)	0.0584 (13)	
H10A	0.4939	0.4811	0.1855	0.070*	
C11	0.4066 (2)	0.6240 (7)	0.1280 (3)	0.0556 (12)	
H11A	0.4182	0.7378	0.1530	0.067*	
C12	0.3500 (2)	0.4037 (7)	0.0506 (2)	0.0470 (11)	
H12A	0.3150	0.3340	0.0114	0.056*	
C13	0.3337 (2)	1.0360 (6)	0.1494 (3)	0.0467 (11)	

### Atomic displacement parameters (Å^2^)

**Table:**

	*U*^11^	*U*^22^	*U*^33^	*U*^12^	*U*^13^	*U*^23^
Mn	0.0374 (5)	0.0426 (5)	0.0373 (5)	0.0014 (5)	0.0210 (4)	0.0017 (5)
S	0.0671 (9)	0.1062 (13)	0.0473 (8)	−0.0215 (8)	0.0295 (7)	−0.0212 (8)
N1	0.048 (2)	0.070 (3)	0.047 (2)	−0.002 (2)	0.0289 (19)	0.012 (2)
N2	0.047 (2)	0.049 (2)	0.042 (2)	0.0012 (18)	0.0277 (17)	0.0060 (19)
N3	0.0365 (19)	0.052 (2)	0.047 (2)	0.0070 (18)	0.0229 (17)	0.0047 (19)
N4	0.040 (2)	0.050 (2)	0.041 (2)	0.0055 (18)	0.0210 (17)	0.0058 (18)
N5	0.056 (2)	0.052 (2)	0.053 (2)	−0.010 (2)	0.030 (2)	−0.011 (2)
C1	0.107 (5)	0.108 (6)	0.130 (6)	−0.023 (5)	0.067 (5)	0.032 (5)
C2	0.116 (5)	0.065 (4)	0.073 (4)	−0.007 (4)	0.043 (4)	0.011 (3)
C3	0.069 (3)	0.081 (4)	0.064 (3)	−0.003 (3)	0.036 (3)	0.023 (3)
C4	0.055 (3)	0.072 (3)	0.056 (3)	−0.001 (3)	0.039 (2)	0.002 (3)
C5	0.059 (3)	0.052 (3)	0.056 (3)	0.004 (2)	0.036 (3)	0.003 (2)
C6	0.049 (3)	0.060 (3)	0.051 (3)	0.007 (2)	0.029 (2)	0.010 (3)
C7	0.093 (4)	0.094 (5)	0.094 (4)	0.008 (4)	0.070 (4)	0.001 (4)
C8	0.057 (3)	0.062 (3)	0.073 (3)	0.008 (3)	0.040 (3)	−0.007 (3)
C9	0.051 (3)	0.052 (3)	0.067 (3)	0.009 (2)	0.032 (3)	0.007 (3)
C10	0.041 (3)	0.070 (4)	0.052 (3)	0.005 (3)	0.020 (2)	−0.006 (3)
C11	0.049 (3)	0.056 (3)	0.054 (3)	−0.001 (2)	0.025 (2)	−0.010 (3)
C12	0.039 (2)	0.055 (3)	0.044 (2)	0.002 (2)	0.022 (2)	0.002 (2)
C13	0.046 (3)	0.048 (3)	0.054 (3)	0.003 (2)	0.033 (2)	0.003 (2)

### Geometric parameters (Å, °)

**Table:**

Mn—N5^i^	2.229 (4)	C1—H1B	0.9300
Mn—N5	2.229 (4)	C2—C3	1.444 (7)
Mn—N2	2.269 (3)	C2—H2A	0.9300
Mn—N2^i^	2.269 (3)	C3—H3A	0.9700
Mn—N4^i^	2.271 (3)	C3—H3B	0.9700
Mn—N4	2.271 (3)	C4—C5	1.342 (6)
S—C13	1.621 (5)	C4—H4A	0.9300
N1—C6	1.345 (5)	C5—H5B	0.9300
N1—C4	1.346 (6)	C6—H6A	0.9300
N1—C3	1.465 (6)	C7—C8	1.279 (7)
N2—C6	1.315 (5)	C7—H7A	0.9300
N2—C5	1.368 (5)	C7—H7B	0.9300
N3—C12	1.348 (5)	C8—C9	1.477 (6)
N3—C10	1.358 (6)	C8—H8A	0.9300
N3—C9	1.460 (6)	C9—H9A	0.9700
N4—C12	1.322 (5)	C9—H9B	0.9700
N4—C11	1.373 (5)	C10—C11	1.342 (6)
N5—C13	1.160 (5)	C10—H10A	0.9300
C1—C2	1.301 (8)	C11—H11A	0.9300
C1—H1A	0.9300	C12—H12A	0.9300
			
N5^i^—Mn—N5	180.0 (2)	N1—C3—H3A	109.0
N5^i^—Mn—N2	91.68 (13)	C2—C3—H3B	109.0
N5—Mn—N2	88.32 (13)	N1—C3—H3B	109.0
N5^i^—Mn—N2^i^	88.32 (13)	H3A—C3—H3B	107.8
N5—Mn—N2^i^	91.68 (13)	C5—C4—N1	106.8 (4)
N2—Mn—N2^i^	180.00 (19)	C5—C4—H4A	126.6
N5^i^—Mn—N4^i^	91.04 (14)	N1—C4—H4A	126.6
N5—Mn—N4^i^	88.96 (14)	C4—C5—N2	110.0 (4)
N2—Mn—N4^i^	89.22 (12)	C4—C5—H5B	125.0
N2^i^—Mn—N4^i^	90.78 (12)	N2—C5—H5B	125.0
N5^i^—Mn—N4	88.96 (14)	N2—C6—N1	111.5 (4)
N5—Mn—N4	91.04 (14)	N2—C6—H6A	124.2
N2—Mn—N4	90.78 (12)	N1—C6—H6A	124.2
N2^i^—Mn—N4	89.22 (12)	C8—C7—H7A	120.0
N4^i^—Mn—N4	180.0	C8—C7—H7B	120.0
C6—N1—C4	106.9 (4)	H7A—C7—H7B	120.0
C6—N1—C3	127.5 (4)	C7—C8—C9	126.7 (5)
C4—N1—C3	125.6 (4)	C7—C8—H8A	116.7
C6—N2—C5	104.8 (4)	C9—C8—H8A	116.7
C6—N2—Mn	128.2 (3)	N3—C9—C8	114.1 (4)
C5—N2—Mn	126.8 (3)	N3—C9—H9A	108.7
C12—N3—C10	106.2 (4)	C8—C9—H9A	108.7
C12—N3—C9	127.0 (4)	N3—C9—H9B	108.7
C10—N3—C9	126.9 (4)	C8—C9—H9B	108.7
C12—N4—C11	104.6 (4)	H9A—C9—H9B	107.6
C12—N4—Mn	125.7 (3)	C11—C10—N3	107.3 (4)
C11—N4—Mn	129.6 (3)	C11—C10—H10A	126.4
C13—N5—Mn	157.6 (4)	N3—C10—H10A	126.4
C2—C1—H1A	120.0	C10—C11—N4	109.9 (4)
C2—C1—H1B	120.0	C10—C11—H11A	125.0
H1A—C1—H1B	120.0	N4—C11—H11A	125.0
C1—C2—C3	125.2 (7)	N4—C12—N3	112.0 (4)
C1—C2—H2A	117.4	N4—C12—H12A	124.0
C3—C2—H2A	117.4	N3—C12—H12A	124.0
C2—C3—N1	113.1 (4)	N5—C13—S	179.4 (5)
C2—C3—H3A	109.0		
			
N5^i^—Mn—N2—C6	81.3 (4)	C4—N1—C3—C2	72.8 (7)
N5—Mn—N2—C6	−98.7 (4)	C6—N1—C4—C5	−0.2 (5)
N4^i^—Mn—N2—C6	172.3 (4)	C3—N1—C4—C5	−179.9 (4)
N4—Mn—N2—C6	−7.7 (4)	N1—C4—C5—N2	0.5 (6)
N5^i^—Mn—N2—C5	−105.3 (4)	C6—N2—C5—C4	−0.5 (5)
N5—Mn—N2—C5	74.7 (4)	Mn—N2—C5—C4	−175.1 (3)
N4^i^—Mn—N2—C5	−14.3 (4)	C5—N2—C6—N1	0.3 (5)
N4—Mn—N2—C5	165.7 (4)	Mn—N2—C6—N1	174.8 (3)
N5^i^—Mn—N4—C12	−8.1 (3)	C4—N1—C6—N2	−0.1 (5)
N5—Mn—N4—C12	171.9 (3)	C3—N1—C6—N2	179.6 (4)
N2—Mn—N4—C12	83.6 (3)	C12—N3—C9—C8	105.3 (5)
N2^i^—Mn—N4—C12	−96.4 (3)	C10—N3—C9—C8	−76.3 (6)
N5^i^—Mn—N4—C11	167.3 (4)	C7—C8—C9—N3	−7.1 (8)
N5—Mn—N4—C11	−12.7 (4)	C12—N3—C10—C11	0.5 (5)
N2—Mn—N4—C11	−101.0 (4)	C9—N3—C10—C11	−178.2 (4)
N2^i^—Mn—N4—C11	79.0 (4)	N3—C10—C11—N4	−0.7 (6)
N2—Mn—N5—C13	67.0 (9)	C12—N4—C11—C10	0.7 (5)
N2^i^—Mn—N5—C13	−113.0 (9)	Mn—N4—C11—C10	−175.4 (3)
N4^i^—Mn—N5—C13	156.3 (9)	C11—N4—C12—N3	−0.4 (5)
N4—Mn—N5—C13	−23.7 (9)	Mn—N4—C12—N3	175.9 (3)
C1—C2—C3—N1	−120.3 (7)	C10—N3—C12—N4	−0.1 (5)
C6—N1—C3—C2	−106.9 (6)	C9—N3—C12—N4	178.6 (4)

Symmetry codes: (i) −*x*+1/2, −*y*+3/2, −*z*.

### Hydrogen-bond geometry (Å, °)

**Table:**

*D*—H···*A*	*D*—H	H···*A*	*D*···*A*	*D*—H···*A*
C7—H7A···N3	0.93	2.54	2.857 (9)	101.
C6—H6A···N4	0.93	2.88	3.355 (8)	113.
C5—H5B···N4^i^	0.93	2.82	3.298 (7)	113.
C12—H12A···N5^i^	0.93	2.72	3.224 (6)	115.

Symmetry codes: (i) −*x*+1/2, −*y*+3/2, −*z*.
